# Monitoring geological storage of CO_2_: a new approach

**DOI:** 10.1038/s41598-021-85346-8

**Published:** 2021-03-15

**Authors:** Manzar Fawad, Nazmul Haque Mondol

**Affiliations:** 1 Department of Geosciences, University of Oslo, Oslo, Norway; 2 Norwegian Geotechnical Institute (NGI), Oslo, Norway

**Keywords:** Geophysics, Carbon capture and storage

## Abstract

Geological CO_2_ storage can be employed to reduce greenhouse gas emissions to the atmosphere. Depleted oil and gas reservoirs, deep saline aquifers, and coal beds are considered to be viable subsurface CO_2_ storage options. Remote monitoring is essential for observing CO_2_ plume migration and potential leak detection during and after injection. Leak detection is probably the main risk, though overall monitoring for the plume boundaries and verification of stored volumes are also necessary. There are many effective remote CO_2_ monitoring techniques with various benefits and limitations. We suggest a new approach using a combination of repeated seismic and electromagnetic surveys to delineate CO_2_ plume and estimate the gas saturation in a saline reservoir during the lifetime of a storage site. This study deals with the CO_2_ plume delineation and saturation estimation using a combination of seismic and electromagnetic or controlled-source electromagnetic (EM/CSEM) synthetic data. We assumed two scenarios over a period of 40 years; Case 1 was modeled assuming both seismic and EM repeated surveys were acquired, whereas, in Case 2, repeated EM surveys were taken with only before injection (baseline) 3D seismic data available. Our results show that monitoring the CO_2_ plume in terms of extent and saturation is possible both by (i) using a repeated seismic and electromagnetic, and (ii) using a baseline seismic in combination with repeated electromagnetic data. Due to the nature of the seismic and EM techniques, spatial coverage from the reservoir's base to the surface makes it possible to detect the CO_2_ plume’s lateral and vertical migration. However, the CSEM low resolution and depth uncertainties are some limitations that need consideration. These results also have implications for monitoring oil production—especially with water flooding, hydrocarbon exploration, and freshwater aquifer identification.

## Introduction

CO_2_ capture, transport, and storage (CCS) is the technology with the potential to significantly prevent CO_2_ build-up in the atmosphere from fossil fuel use. The oil and gas industry has been injecting gases, including CO_2_, to dispose of the non-commercial components of the produced hydrocarbon, and for enhanced hydrocarbon recovery^1,2^. Besides, natural gas on a large scale is stored in many parts of the world for load management related to user's demand^3^. Even though subsurface accumulations of CO_2_ occur naturally in many parts of the world, large-scale human-made CO_2_ storage still poses various technical and social challenges^4^.

One of the concerns is detecting the CO_2_ plume migration (for example, in a saline aquifer) and possible leakage. Among the techniques suggested today for remote CO_2_ monitoring are repeat 3D seismic (also called 4D, or time-lapse seismic), repeat electromagnetic surveys (4D EM/CSEM), microseismic, InSAR, and tiltmeter/GPS monitoring. The seismic has been identified as a high-cost, high-benefit, whereas the EM is considered high-cost, low-benefit CO_2_ monitoring techniques on Boston square matrix^1^. The Boston square matrix helps decision-makers allocate resources and is used as an analytical tool in strategic management and portfolio analysis. Considering a 3D survey area, a node-based CSEM is equally priced or slightly cheaper than a towed 3D seismic survey. However, the CSEM is underused today. The technology is still in a developing stage, and one can expect a reduction in CSEM cost with an increase in its usage and new technology implementation.

We put forward a novel approach combining seismic and electromagnetic (EM/CSEM) information to monitor plume in the subsurface for lateral and vertical migration, with the additional benefit of CO_2_ saturation estimation. The proposed technique, despite the high cost, will be valuable in terms of enhanced and reliable control on the CO_2_ injection and storing processes.

**Figure 1:**
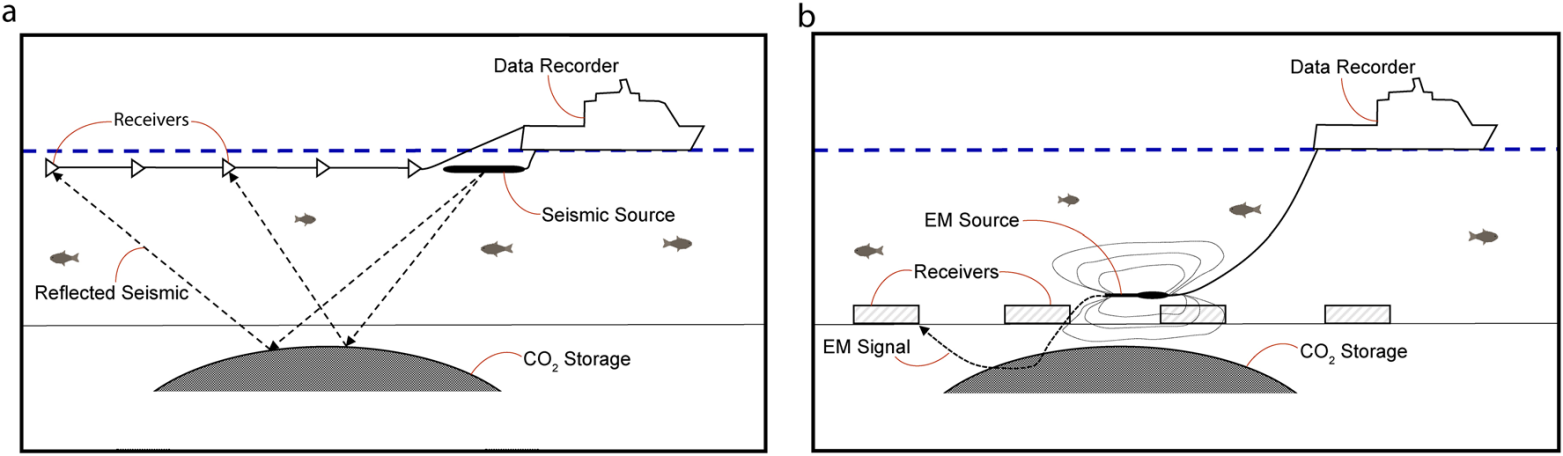
(**a**) Seismic and (**b**) electromagnetic acquisition methods in the case of marine setup.

Seismic data acquisition is routinely performed both on land and at sea. Seismic vessels deploy one or more cables (streamers) behind it as they move forward at sea. Each streamer includes multiple receivers in a configuration (Fig. 1a). Streamer trails behind a vessel, which moves forward as the survey progresses. The seismic source is also towed behind the vessel. Source and receivers are typically deployed below the surface of the sea. Data transmitted to the ship through cables is recorded and processed. The source emits seismic waves that reflect from formation boundaries. The reflected waves are detected by receivers and recorded as a function of time by determining the time it takes for seismic waves to propagate from source, reflected at a boundary, and back to receivers. The recorded signal may yield the position's information, the topography of boundary, rock, and in-situ fluid properties. The receivers used in marine seismology are commonly referred to as hydrophones or marine pressure phones. When used in combination with other available geophysical, borehole, and geological data, seismic surveys provide useful information about the structure and distribution of subsurface rock properties and their interstitial fluids^5,6^. One of the outcomes we get from the inversion of seismic data is the acoustic impedance (AI), which is the multiplication of acoustic P-wave velocity and the rock’s bulk density. Oil companies employ interpretation of such seismic data for selecting the sites to drill oil and gas exploratory and development wells.

The acquisition and inversion of electromagnetic data have, in recent years, become a valuable tool in investigating potential hydrocarbon-bearing formations. In most Controlled-source electromagnetic (CSEM) surveying acquisitions, a system comprises an electromagnetic emitter or antenna that is either pulled from a vessel, stationary in the body of water or on the seabed, and likewise a number of electromagnetic receivers that are fixed on the seabed or towed from a vessel or stationary in the body of water (Fig. 1b). The receivers record variations in electrical resistance depending on variations in source signal, offset between the source and receiver, and rock layers' geological properties, including their inherent electrical resistivity properties. For instance, a CO_2_-bearing layer will exhibit a higher electrical resistivity than the seawater or overburden of sediments. The CSEM Inversion techniques, which yield vertical and horizontal resistivity, have been developed to optimize a model's parameters to find the best fit between the calculated value and the measured data while constraining the model employing the measured data.

When borehole logs are available from nearby wells, seismic survey and CSEM data can be enhanced and calibrated using the log data. Compared to the CSEM resistivity, the log resistivity measured is usually assumed to be the horizontal component, primarily due to the borehole tool's design. Here for simplicity we assume the rock's physical properties are homogeneous in all directions (isotropic). Instead of splitting into vertical and horizontal components, we will use the general term resistivity. The existing methods are based on applying resistance directly from CSEM inversion results and inserting these into an appropriate saturation-resistivity relation, such as Archie's equation^7^ or similar. Using porosity derived from Wyllie’s equation^8^ applied on the seismic-derived velocity, total resistivity from the CSEM, and assuming the water resistivity are known, the fluid saturation (S_fl_) estimate can be obtained^9,10^.

One may use different mixtures theories to obtain the electromagnetic and seismic properties and then combine them in different ways. For instance, Archie’s law or the complex refraction-index method (CRIM) combined with the time-average equation are two possible choices^10^. Other techniques involve relating the Gassmann equation^11^ with the different electromagnetic related equations. Further possibilities involve the Hashin–Shtrikman upper and lower bounds^12^ and the self-similar equation. In the case of plane-layered composites, Backus averaging to relate the conductivity and stiffness tensors can be considered, where the common property is the material proportion^10^.

**Figure 2:**
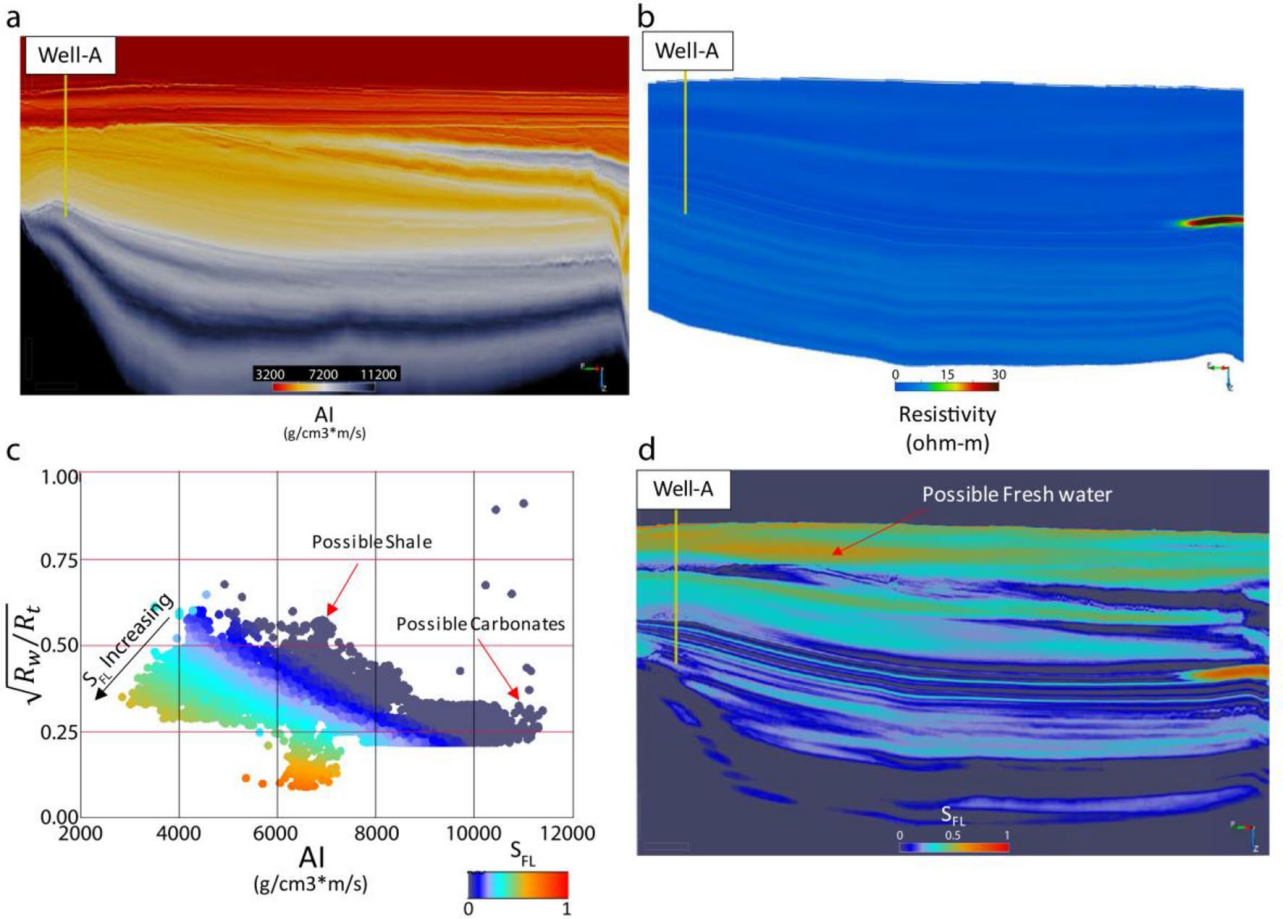
An example of fluid (oil in this case) response using synthetic data, (**a**) AI profile with a dry well (Well-A), (**b**) An oil accumulation on extreme west exhibiting an anomaly on the resistivity section that is not obvious on the AI profile (**a**), (**c**) The AI-Resistivity ratio function ($\sqrt{{R}_{w}/{R}_{t}}$) plane showing that the fluid effect can be isolated and quantified using our proposed technique, (**d**) the resulting fluid saturation profile indicating oil anomaly and possible freshwater.

As mentioned previously, the AI is obtained by inverting seismic data. AI increases typically with increasing compaction as a result of a decrease in porosity. If the target fluid (e.g., oil or freshwater) has an identical density as the in-situ saltwater, the change in acoustic impedance will be insignificant (Fig. 2a). On the other hand, if the target fluid has a low density like CO_2_ or hydrocarbon gases, a noticeable decrease in acoustic impedance is expected. In a reservoir, the in-situ salt content in brines makes the total resistivity of a reservoir very low; however, the presence of hydrocarbon, freshwater, or injected CO_2_ increases the overall resistivity of the reservoir, making it possible to detect this change using the CSEM method (Fig. 2b). On the other hand, the reduction of porosity due to rock compaction also increases total resistivity, which needs to be differentiated from fluid-related resistivity. There had been a necessity to directly relate acoustic impedance with the resistivity with an ability to calibrate locally, considering the rock matrix and in-situ conditions using borehole data. We came up with an equation (see “Methods” section) that relates AI with resistivity to isolate the salt water-bearing rock compaction trend and the target fluid saturation (Fig. 2c,d). One can estimate the resistivity of water (R_w_) from a nearby well (Well-A in this case).

**Figure 3:**
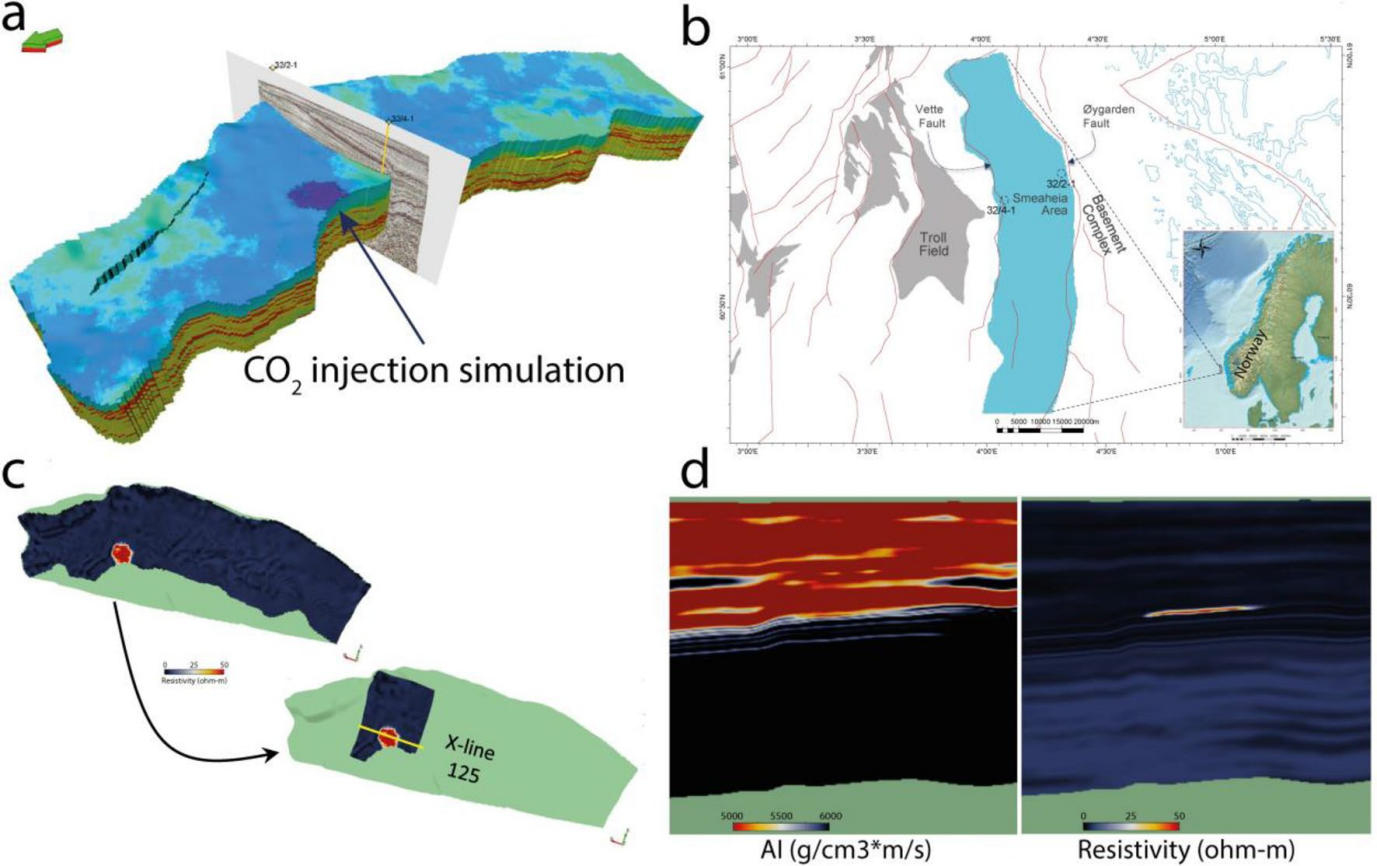
(**a**) Initial simulation modeling grid used for property extraction, (**b**) location of the modeled grid area (light blue) in the Norwegian North Sea, maps modified after NPD^15^, (**c**) example of resistivity grid carved out to a seismic formatted cube covering only the injection and storage area, (**d**) AI and resistivity profiles along crossline 125 shown in (**c**), the example here is of the year 2050, the effect of injected CO_2_ on AI is not very obvious, however on resistivity profile one can see a significant anomaly.

For the present study, we acquired synthetic data from the Norwegian Geotechnical Institute (NGI). NGI generated AI and Resistivity properties^13^ using grids from a reservoir model by the Northern Light project^14^ (Fig. 3a). The model simulated one of the potential CO_2_ storage sites in the northern North Sea called Smeaheia (Fig. 3b). The amount of CO_2_ considered sequestering was 1.3 MT/Year employing an injection period of 25 years with an injection rate of 200 tons/hr. The Smeaheia area is bounded by a fault array separating the Troll oil and gas field in the west and the Basement Complex in the east. Sognefjord Formation (Upper Jurassic) sandstone is the main CO_2_ storage reservoir in the Smeaheia area, capped by the Draupne and Heather Formation (Upper Jurassic) shales. We carved out the AI and Resistivity cubes covering only the injection and storage area for fast digital handling, converting to a depth-domain seismic format with inline and crossline profiles (Fig. 3c). We assumed that the AI and Resistivity cubes are the actual values obtained from the seismic and CSEM data inversion (Fig. 3d).

We assumed two monitoring scenarios over 40 years, with injection starting in 2020 for 25 years. In one scenario, i.e., Case 1, the assumption was that both seismic and EM repeated surveys were acquired every 10 years. To cut the monitoring cost (Case 2), only baseline 3D seismic data was acquired in 2020 (before CO_2_ injection), with repeated EM surveys taken every 10 years until 2060. This study also has implications for hydrocarbon exploration, freshwater aquifer identification, and monitoring of oil production—especially with water flooding. The physical properties like anisotropy, CO_2_ dissolution, and chemical reaction with rock grains and their effect on the AI and resistivity are not taken into account. The EM low resolution and depth uncertainties are some limitations, which warrant consideration.

## Results and discussion

### Case 1

**Figure 4:**
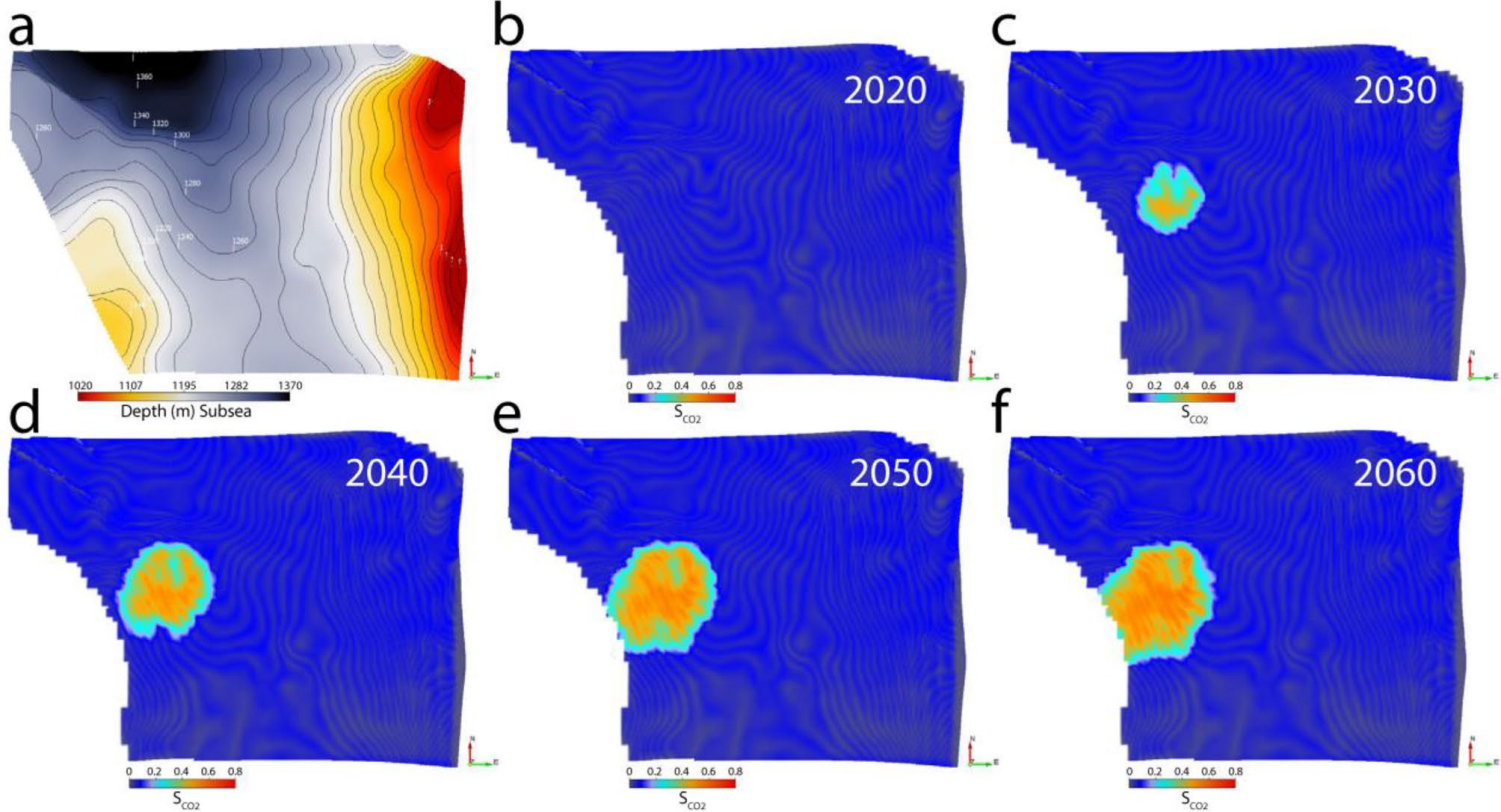
The top reservoir depth surface (**a**) draped on Case 1 CO_2_ saturation cubes in year (**b**) 2020, (**c**) 2030, (**d**) 2040, (**e**) 2050, and (**f**) 2060. The CO_2_ plume moves up-dip over time towards the southwest.

In this scenario, we have both seismic and EM data from 2020 before injection to the year 2060. The reservoir AI decreases where the CO_2_ plume reaches, whereas the resistivity increases as the CO_2_ replaces the conductive saltwater residing within the pores. Therefore, the estimated saturations from AI and resistivity supposedly inverted from the seismic and EM respectively very well define the plume boundaries and reservoir inhomogeneity (Fig. 4). We can also see the plume boundary systematically increasing with the passage of years and tends to move towards the southwest in the up-dip direction (Fig. 4).

### Case 2

**Figure 5:**
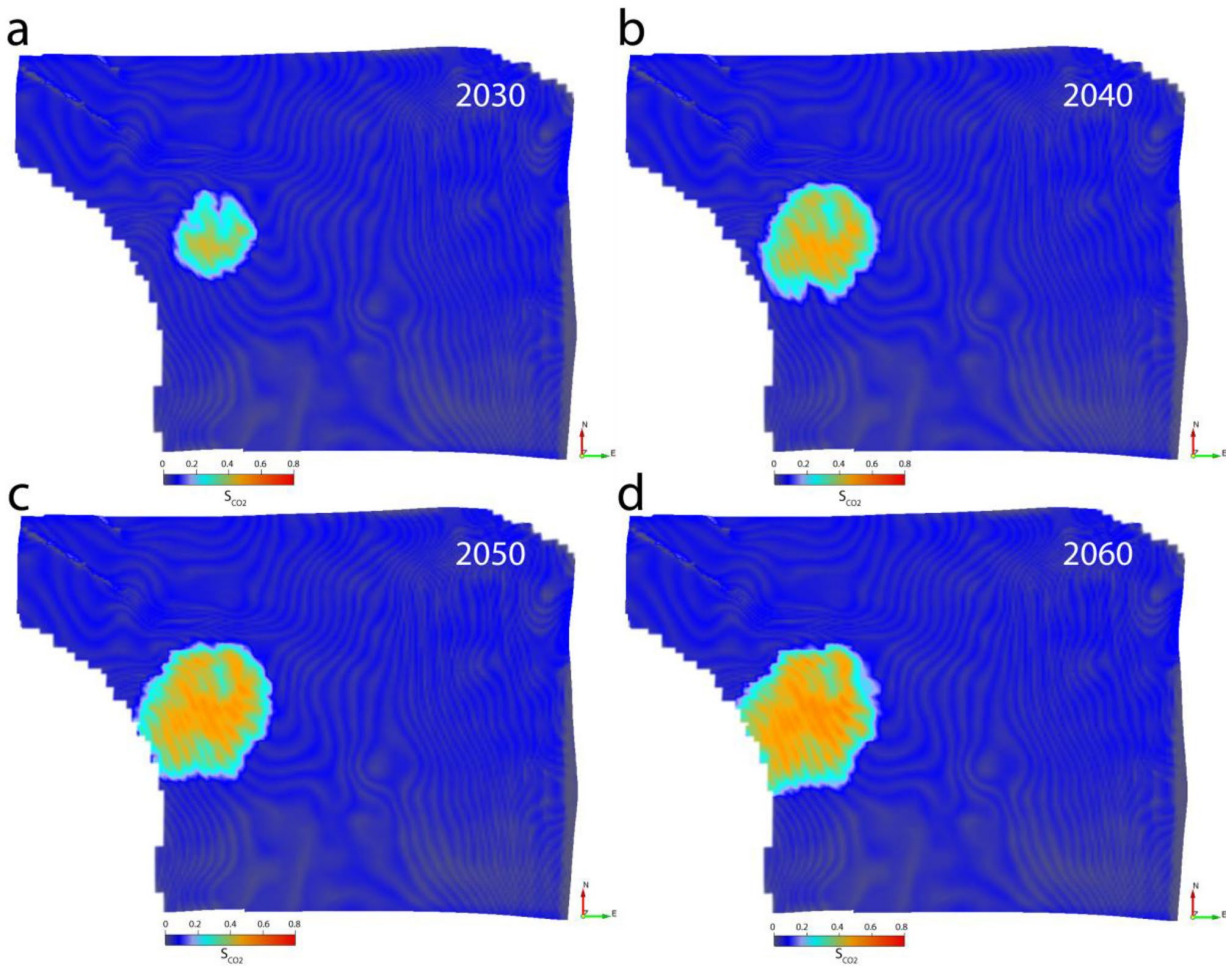
Case 2 results: the top reservoir depth surface draped on CO_2_ saturation cubes in (**a**) 2030, (**b**) 2040, (**c**) 2050, and (**d**) 2060. The CO_2_ plume area is identical to that of Case 1, with apparently slight under-prediction in saturation.

This case addresses the reduction of monitoring cost scenario assuming only the baseline seismic survey (in the year 2020) and repeated EM survey every 10 years until 2060 (Fig. 5). Here we assume that the CO_2_ plume is not changing the reservoir’s AI values, whereas resistivity increases where the plume reaches. Theoretically, we can expect an increase in saturation estimation accuracy if the displacement fluid’s density and P-wave velocity are roughly equal to that of the displaced fluid. But even supersaturated CO_2_ density and P-wave velocity are low compared to that of the saltwater; therefore, we can expect a slight under-prediction of CO_2_ saturation in this case.

### The difference between the estimated saturation of Case 1 and Case 2

**Figure 6:**
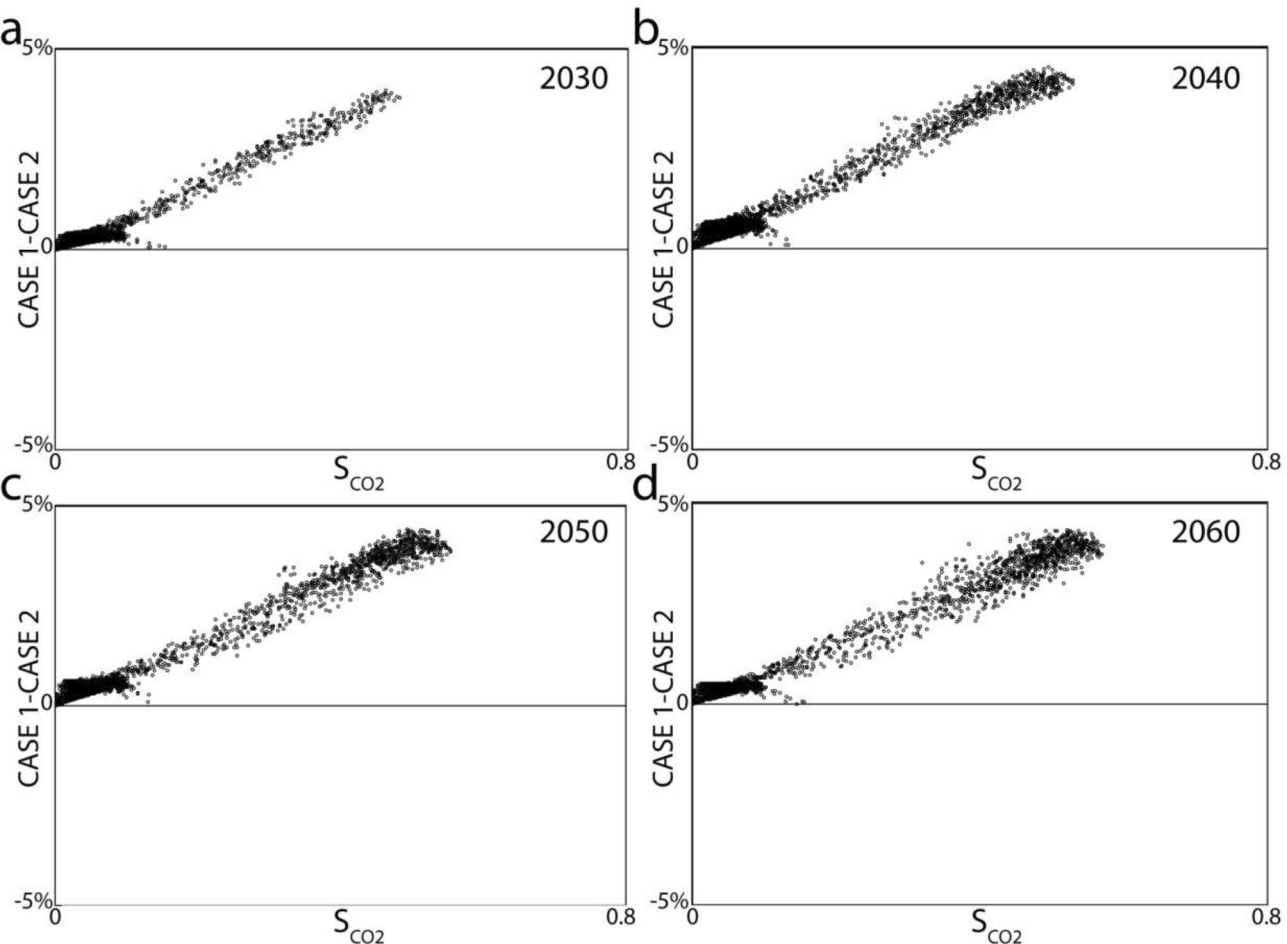
The difference of saturation estimation between Case 1 and Case 2 is plotted against the saturation obtained in Case 1 for years (**a**) 2030, (**b**) 2040, (**c**) 2050, and (**d**) 2060 accordingly. Data points were sampled at regular intervals on the top Sognefjord surface, from which 50% are displayed on the crossplots.

We calculated the CO_2_ saturation difference between the two cases by subtracting the Case 2 saturation cube from Case 1 saturation of the respective year. It is revealed that the saturation estimation difference between the two cases increases with an increase in saturation (Fig. 6). The estimation difference, however, is less than 5%. This implies that using a baseline seismic with repeated CSEM can effectively be used for CO_2_ monitoring with slight under-prediction of saturation. This under-prediction (< 5%) we deem is within acceptable limits. We infer that Case 2 has the potential to yield accurate saturation estimation if water flooding is used to displace oil in an enhanced oil recovery scenario since the density and P-wave velocity values of oil are close to that of saltwater.

### Depth and saturation sensitivities

The EM method's structural resolution is poor, and without further constraint, the depth of objects of interest may be uncertain. As the EM wavelengths are much longer than seismic wavelengths, therefore, the vertical EM resolution is significantly lower than the vertical seismic resolution^9^. The penetration depth (d) for an EM field in conductive media is:

1 $$d = 503\sqrt{\frac{R}{f}}$$

where R is the rock resistivity, and f is EM frequency. It is obvious from Eq. 1 that using high frequencies will reduce the penetration depth. On the other hand, lowering the frequencies will deteriorate the resolution. Therefore, it is crucial to use a sufficiently broad frequency range to improve the vertical resolution. The noise level and receiver spacing mainly limit the lateral resolution of the EM data. The attenuation of EM energy with depth restricts the use of the technology. With the current source strengths, it is challenging to reach depths of more than 3 km below the seabed due to lower resolution and higher noise level than the real signal^16^. For subsurface storage, CO_2_ is injected in its dense (supercritical) phase to a depth where the temperature and pressure keep the gas in the same phase. This strategy maximizes the use of available storage volume in the pore spaces within a reservoir. Therefore, the optimum depth for storage is between 1 and 3 km depth^4^, which is an appropriate depth range for implementing the CSEM technology. Furthermore, the resistivity of a saline aquifer containing CO_2_ is dependent on CO_2_ saturation, and the CSEM measurements will have appreciable sensitivity to increasing saturation changes compared to the seismic velocity, especially in the mid-to-high saturation ranges^17^. So our proposed approach could prove suitable for CO_2_ storage monitoring provided the depth and resolution issues are addressed in the EM inversion.

### Other suggested usage of the technique

We can see the potential to explore freshwater in the regions where there is an acute water shortage. The freshwater owes high resistivity. This property makes it possible to detect a freshwater accumulation using EM-derived resistivity with the combination of the AI inverted from seismic. In the future similar setup to identify subsurface water on other planets is also possible. However, in places, the presence of conductive clays within the reservoir might limit the procedure’s application. There is a problem of saltwater intrusion into freshwater aquifers in many coastal areas^18,19^, leading to groundwater quality degradation, including drinking water sources, and other consequences. Saltwater intrusion occurs naturally in coastal aquifers, owing to the hydraulic connection between groundwater and seawater. Monitoring the freshwater-saltwater interface, in that case, can be performed with our suggested technique.

Oil has high resistivity; however, depending on viscosity, the oil density and P-wave velocity might be roughly similar to that of the water. Therefore, the time-lapse seismic will not detect significant AI changes during oil production using water flooding. Using the EM-seismic combination for oil-production monitoring will be invaluable in that case.

## Conclusions

The seismic method is in most situations, provides high-resolution images of structure and stratigraphy. Seismic data can be inverted to provide quantitative information on physical properties such as acoustic impedance (AI); however, seismic itself is not sufficient to discriminate fluids and their saturations in many situations. Electrical resistivity is well known to respond sharply to changing fluid type and saturation. The Controlled Source Electro-Magnetic (CSEM) technique measures resistivity from the seafloor, which can also be ambiguous if taken alone. The method's structural resolution is low, and without further constraint, the depth of features of interest may be uncertain. Nor can high resistivity zones be linked explicitly to a target fluid (i.e., CO_2_, hydrocarbon, or freshwater): they could equally be caused by tight sands or carbonates, salt, or volcanic intrusions, among other things.

We combined the information obtained from the CSEM and seismic inversion within a rock physics framework to resolve many ambiguities mentioned above. We demonstrated using a model that the complementary measurement of resistivity derived from CSEM helps predict CO_2_ saturation in a reservoir during and after injection in a subsurface geological CO_2_ storage.

Modeling using our proposed approach showed that CO_2_ saturation estimation and plume area delineation is possible using acoustic impedance (AI) from a baseline seismic and resistivity from repeated electromagnetic (Case 2). However, information from both repeated seismic and electromagnetic (Case 1) will increase the estimation accuracy. The Saturation difference between the two cases (Case 1 and 2) increased with increasing saturation; however, the difference was not more than 5%.

One can also use the suggested procedure to monitor oil production using water flooding, finding and monitoring freshwater aquifer, and hydrocarbon exploration. The CSEM technology is underused; therefore, it is expensive. The EM acquisition and inversion procedures are still in the development stages. We expect the combined usage of the seismic and EM will increase significantly with progress in efficient technologies, making the method relatively inexpensive in the future.

## Methods

**Figure 7:**
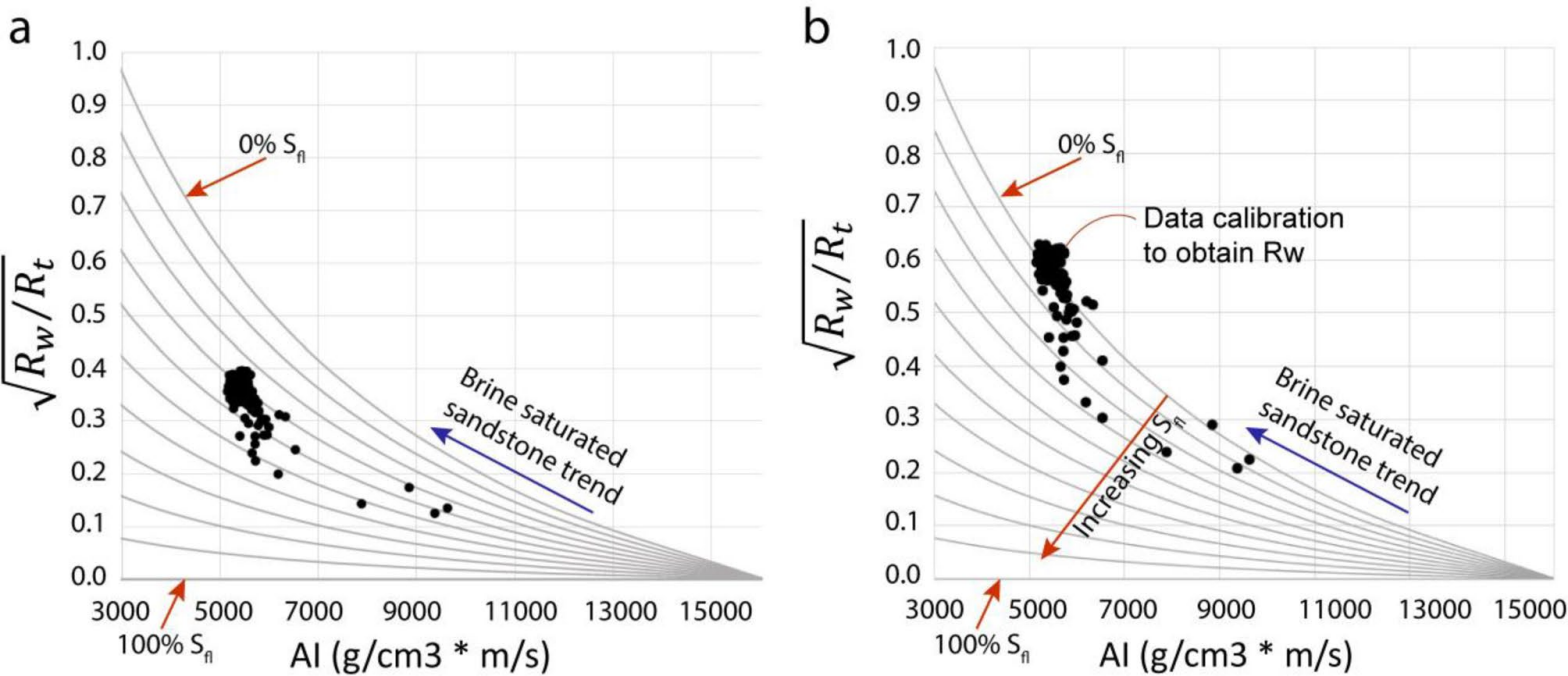
Method of finding R_w_ by calibrating the brine sandstone/siltstone trend in the data with the reference 0% fluid saturation (S_fl_) line onto the Acoustic impedance-resistivity ratio function ($\sqrt{{R}_{w}/{R}_{t}}$) plane. (**a**) Data before calibration, (**b**) data after R_w_ iteration and adjustment, so the brine saturated sandstone trend is calibrated with the 0% S_fl_ line.

We generated a rock physics model assuming that a reservoir consists of a rock matrix, pore spaces containing saltwater, and other fluids (e.g., CO_2_, hydrocarbon, or freshwater). An acoustic impedance (AI) baseline on the x-axis was defined with end members from the matrix (e.g., quartz) to the target fluid. This baseline was assumed to be having infinite resistivity and zero porosity. According to the assumption, the total volume of rock comprising the matrix and the fluids in the pore spaces is equal to 1. Wyllie^8^ approximated the relation between velocity and volumes in sedimentary rocks with this expression:

2 $$\frac{1}{{\mathrm{V}}_{\mathrm{P}}}=\frac{(1-\varnothing )}{{\mathrm{V}}_{{\mathrm{P}}_{\mathrm{ma}}}}+\frac{{\mathrm{S}}_{\mathrm{fl}}\varnothing }{{\mathrm{V}}_{{\mathrm{P}}_{\mathrm{fl}}}}+\frac{(1-{\mathrm{S}}_{\mathrm{fl})}\varnothing }{{\mathrm{V}}_{{\mathrm{P}}_{\mathrm{w}}}}$$

where Vp, Vp_ma_, Vp_fl_, and Vp_w_ are the P-wave velocities of the saturated rocks, the rock matrix, the pore fluid (other than saltwater), and saltwater (brine), respectively, $\mathrm{\varnothing }$ is pore space volume. This equation is often called the time-average equation. It is heuristic and cannot be justified theoretically; however, it is useful for estimating P-wave velocity directly without going into the elastic moduli components. The bulk density (ρ_b_) is a volumetric average of the densities of the rock constituents that can be related to the various rock volume components by:

3 $${\uprho }_{\mathrm{b}}=\left(1-\mathrm{\varnothing }\right){\uprho }_{\mathrm{ma}}+ {S}_{fl}\varnothing {\uprho }_{\mathrm{fl}}+ \left(1-{S}_{fl}\right){\mathrm{\varnothing \rho }}_{\mathrm{w}}$$

where ρ_ma_ is the density of rock grains, ρ_fl_ is the density of pore fluid other than saltwater, ρ_w_ is the density of saltwater (brine). In a fluid-saturated rock, the total resistivity (R_t_) of rock according to Archie’s equation^7^ is:

4 $${\mathrm{R}}_{\mathrm{t}}= \left(\frac{\mathrm{a}}{{\left({1-S}_{fl}\right)}^{2}{\varnothing }^{2}}\right){\mathrm{R}}_{\mathrm{w}}$$

where R_w_ is the resistivity of saltwater, and a is the "tortuosity factor". Combining Eqs. (2), (3), and (4), we obtain:

5 $$\sqrt{\frac{{R}_{w}}{{R}_{t}}}= \frac{\left({\rho }_{ma} -\frac{AI}{ {V}_{{P}_{ma}}}\right)\left(1-{S}_{fl}\right)}{\sqrt{a }\left[AI\left\{{S}_{fl}\left(\frac{1}{{V}_{Pfl}}-\frac{1}{{V}_{Pw}}\right)+\left(\frac{1}{{V}_{Pw}}-\frac{1}{{V}_{Pma}}\right)\right\}-\left\{{S}_{fl}\left({\rho }_{fl}-{\rho }_{w}\right)+\left({\rho }_{w}-{\rho }_{ma}\right)\right\}\right]}$$

where AI is acoustic impedance, and ($\sqrt{{R}_{w}/{R}_{t}}$) can be named as "resistivity ratio function". The tortuosity factor ‘a’ controls the slope of the iso-saturation curved lines and may be selected in a formation zone depending on pore structure, grain size, and compaction level. The relevant constants may be taken from literature^20^ and vendor's logging chart books. From this function (Eq. 5), we are able to define a set of lines representing different fluid saturations converging at the 100% matrix pole onto the Acoustic impedance-resistivity ratio function plane (Fig. 7).

Now plotting the data using an initial value of R_w_ in this template and rearranging the equation, the fluid saturation can be calculated in fraction (that can be converted to a percentage by multiplying with 100) using the following equation:

6 $${S}_{fl}=\frac{\left[{\rho }_{ma}-AI\left(\frac{1}{{V}_{Pma}}\right)+\sqrt{\frac{a{R}_{w}}{{R}_{t}} }\left\{\left({\rho }_{w}-{\rho }_{ma}\right)-AI\left(\frac{1}{{V}_{Pw}}-\frac{1}{{V}_{Pma}}\right)\right\}\right]}{\left[\sqrt{\frac{a{R}_{w}}{{R}_{t}} }\left\{AI\left(\frac{1}{{V}_{Pfl}}-\frac{1}{{V}_{Pw}}\right)-\left({\rho }_{fl}-{\rho }_{w}\right)\right\}+\left\{{\rho }_{ma}-AI\left(\frac{1}{{V}_{Pma}}\right)\right\}\right]}$$

Since the R_w_ is unknown, the procedure is to iterate the value of R_w,_ making the upper part of the data (representing the brine saturated matrix) to fall on the 0% S_fl_ (Fig. 7a,b).
